# Is there a survival benefit from adjuvant chemotherapy for patients with liver oligometastases from colorectal cancer after curative resection?

**DOI:** 10.1186/s40880-018-0298-8

**Published:** 2018-05-29

**Authors:** Zhizhong Pan, Jianhong Peng, Junzhong Lin, Gong Chen, Xiaojun Wu, Zhenhai Lu, Yuxiang Deng, Yujie Zhao, Qiaoqi Sui, Desen Wan

**Affiliations:** Department of Colorectal Surgery, Sun Yat-sen University Cancer Center, State Key Laboratory of Oncology in South China, Collaborative Innovation Center for Cancer Medicine, 651 Dongfeng Road East, Guangzhou, 510060 Guangdong P. R. China

**Keywords:** Colorectal cancer, Oligometastases, Adjuvant chemotherapy, Liver resection, Benefit

## Abstract

**Background:**

Although colorectal oligometastases to the liver can potentially be cured with aggressive local ablation, the efficacy of adjuvant chemotherapy (ACT) for such metastasis remains unclear. The present study explored the effects of ACT on patients with colorectal liver oligometastases (CLO) after curative resections and aimed to identify patients who could benefit from ACT.

**Methods:**

We retrospectively analyzed 264 eligible patients with CLO who underwent curative resection between September 1999 and June 2015. Recurrence-free survival (RFS) and overall survival (OS) were analyzed using the Kaplan–Meier method and log-rank test; prognostic factors were a by Cox regression modeling.

**Results:**

Among 264 patients, 200 (75.8%) patients received ACT and 64 (24.2%) did not receive ACT. These two groups did not significantly differ in clinicopathologic characteristics, and had comparable 3-year OS and RFS rates (RFS: 42.1% vs. 45.7%, *P *= 0.588; OS: 69.7% vs. 62.7%, *P *= 0.446) over a median follow-up duration of 35.5 months, irrespective of preoperative chemotherapy. ACT markedly improved 3-year OS in high-risk patients with Memorial Sloan-Kettering Cancer Center clinical risk scores (MSKCC-CRS) of 3–5 (68.2% vs. 33.8%, *P* = 0.015), but presented no additional benefit in patients with MSKCC-CRS of 0–2 (72.2% vs. 78.6%, *P* = 0.834). In multivariate analysis, ACT was independently associated with improved OS in patients with MSKCC-CRS of 3–5.

**Conclusions:**

ACT might offer a prognostic benefit in high-risk patients with CLOs after curative liver resection, but not in low-risk patients. Therefore, patients’ risk status should be determined before ACT administration to optimize postoperative therapeutic strategies.

## Introduction

The liver is the most common site of metastasis in patients with colorectal cancer (CRC). At diagnosis, approximately 25% of patients present with synchronous metastases, and approximately 50% patients ultimately develop metachronous metastases [1, 2]. Liver resection is the most effective curative treatment for patients with CRC liver metastasis, with a 5-year survival rate of 40%–50% [3, 4]. However, ~ 60% of patients develop recurrent liver metastases after initial liver resection [5, 6]. Because of this high recurrence rate, adjuvant chemotherapy (ACT) has been investigated for patients with CRC metastasis to the liver. Although several studies have indicated the potential efficacy of ACT in prolonging survival, its benefits had not been definitively shown until now [7–9].

The latest version of the European Society for Medical Oncology Guidelines highlights oligometastatic disease—a disease state that links localized and systemic disease [2]. Notably, oligometastatic disease confined to the liver is potentially curable. Aggressive locally ablative treatments, including liver resection, may prolong survival of patients with colorectal liver oligometastasis (CLO), with a 5-year overall survival (OS) rate of 45.9% as shown in our previous study [10]. Because complete resection is technically easy to perform, with usually good oncologic outcomes, the suitability of ACT for patients with CLO is unclear [2, 11]. Additionally, even among CRC patients with the same disease stage, ACT benefits are determined by such characteristics as preoperative carcinoembryonic antigen (CEA) level, need for emergent surgery, lymphovascular invasion, T stage and lymph node metastasis [12, 13]. To our knowledge, the value of ACT has not been reported for patients who develop CLO after curative resection.

Therefore, the present study explored whether ACT had a survival benefit for patients with CLO who had undergone curative liver resections, with particular respect to patients’ risk classification according to the Memorial Sloan-Kettering Cancer Center clinical risk score (MSKCC-CRS) [14].

## Patients and methods

### Patient selection

We reviewed medical records of consecutive patients with CRC liver metastases who underwent liver resection between September 1999 and June 2015 at Sun Yat-sen University Cancer Center, China. Patients were included in the present study according to the following criteria: (1) histologically confirmed colorectal adenocarcinoma; (2) preoperative metastases confined to the liver; (3) no more than 5 liver metastases; (4) R0 resection for both primary and metastatic tumors; and (5) a minimum follow-up duration of 3 months. Tumor stage was classified according to the 2010 American Joint Committee on Cancer staging system. Eligible patients’ clinicopathologic data and treatment information were reviewed using an electronic medical record system. All procedures were performed according to the ethical standards of the World Medical Association Declaration of Helsinki of 2013. We obtained approval from the independent ethics committee at Sun Yat-sen University Cancer Center, and requested the informed consents before initial treatments.

### Patient treatments

The treatment strategy for every patient in the current study was determined by a multidisciplinary team (MDT) as previously described [15]. Preoperative (neoadjuvant) chemotherapy (NAC) and ACT regimens were determined based on evaluations by oncologists, and included XELOX (130 mg/m^2^ intravenous [i.v.] oxaliplatin on Day 1 and 1000 mg/m^2^ oral capecitabine twice daily on Days 1–14 for a 3-week cycle), FOLFOX (85 mg/m^2^ i.v. oxaliplatin and 400 mg/m^2^ i.v. leucovorin [LV] on Day 1; 400 mg/m^2^ i.v. 5-fluorouracil (5-FU) on Day 1 and then 1200 mg/m^2^ i.v. 5-FU for Day 1–2 for a 2-week cycle), FOLFIRI (180 mg/m^2^ i.v. irinotecan and 400 mg/m^2^ i.v. LV on Day 1; 400 mg/m^2^ i.v. 5-FU on Day 1 and then 1200 mg/m^2^ i.v. 5-FU for Day 1–2 for a 2-week cycle) and capecitabine (1000 mg/m^2^ oral capecitabine twice daily on Days 1–14 for a 3-week cycle). During NAC, tumor response was assessed using computerized tomography (CT) or magnetic resonance imaging (MRI), by Response Evaluation Criteria in Solid Tumors, version 1.1 [16]. Patients underwent non-anatomical hepatectomy with R0 resection (tumor-free margin > 1 mm). Decisions to use ACT were based on patients’ tolerances and preferences, and was recommended to begin 4–6 weeks after liver resection. Among patients who underwent NAC, their ACT regimens were consistent with NAC.

### Risk status assessment

Recurrence risk in patients after liver resection was evaluated by the MSKCC-CRS [14]. The scoring system is based the following 5 clinical factors: (1) node-positive primary tumor, (2) largest metastasis > 5 cm, (3) multiple liver metastases, (4) preoperative CEA level > 200 ng/mL, and (5) disease-free interval from primary tumor resection to the diagnosis of liver metastasis < 12 months. Based on the number of the risk factors, patients were classified into six risk subgroups (MSKCC-CRS 0–5). Patients with a MSKCC-CRS of 0–2 were classified into the low-risk subgroup, while patients with a MSKCC-CRS of 3–5 were classified into the high-risk subgroup.

### Follow-up

Follow-up data were collected from a tracking system. Patients were monitored through subsequent visits every 3 months for the first 2 years and then semiannually for 5 years after liver resection. Evaluations included clinical examination and assessment of CEA and carbohydrate antigen (CA) 19-9 levels, and CT imaging of the chest, abdomen and pelvis at 3, 6, 12, and 18 months, 2 years, and annually thereafter. Liver MRI was used to confirm suspicious lesions indicated on CT or in patients with increased CEA or CA19-9 level but negative CT results. The final follow-up visit occurred in June 2017. OS was defined as the interval from liver resection to the date of death from any cause or the date of last follow-up. Recurrence-free survival (RFS) was defined as the interval from liver resection to the date of disease recurrence, death from disease or last follow-up. Random censoring was applied to patients without recurrence or death at the last follow-up date. Early recurrence was defined as disease recurrence or death within 6 months after liver resection, and late recurrence was defined as disease recurrence or death at least 6 months after liver resection [17, 18].

### Statistical analysis

All statistical analyses were performed using Statistical Package for the Social Sciences (SPSS, version 21.0, Chicago, IL, USA) and GraphPad Prism version 6.01 (GraphPad, Inc., USA). Values are shown as median (range) or percentage. Continuous and categorical data were compared with the Mann–Whitney U-test, and the Chi square test or Fisher’s exact test, respectively, as appropriate. OS and RFS rates were estimated with the Kaplan–Meier method; differences between groups were assessed with the log-rank test. Parameters for which *P* < 0.10 for OS in univariate Cox models were included in multivariate Cox models. Hazard ratios (HRs) and 95% confidence intervals (CIs) were subsequently calculated. *P* < 0.05 (two-sided) was considered significant.

## Results

### Patient characteristics

We reviewed data from 365 patients with CRC liver metastases who underwent liver resections. After excluding patients with extrahepatic disease or incomplete resections, 283 patients were identified for careful review. We then excluded 17 patients with more than 5 liver metastases and 2 patients with follow-up of less than 3 months for a final study cohort of 264 patients. They included 171 (64.8%) men and 93 (35.2%) women, with a median age of 57 years (range 25–85 years). Their primary tumors were located in the colon for 163 (61.7%) patients and rectum for 101 (38.3%) patients (Table 1). Overall, 171 (64.8%) patients had synchronous metastases at the time of diagnosis. Of the 225 (85.2%) patients for whom MSKCC-CRS could be evaluated, 162 (72.0%) were low-risk (MSKCC-CRS 0–2), and 63 (28.0%) were high-risk (MSKCC-CRS 3–5). In total, 122 (46.2%) patients received NAC, including 47 (38.5%) who received FOLFOX, 32 (26.2%) who received XELOX, 36 (29.5%) who received FOLFIRI, and 7 (5.7%) who received capecitabine. Additionally, 200 (75.8%) patients received ACT, including 57 (28.5%) who received FOLFOX, 82 (41.0%) who received XELOX, 46 (23.0%) who received FOLFIRI, and 15 (7.5%) who received capecitabine. The median duration of ACT was 3.0 months (range 1.0–6.0 months).

**Table 1:** Clinicopathologic characteristics of patients with colorectal oligometastasis to the liver after curative liver resection

Parameters	Total (n)	With ACT (n, %)	Without ACT (n, %)	*P* value
Number of patients	264	200	64	
Age, years				
≤ 60	164	129 (64.5)	35 (54.7)	0.159
> 60	100	71 (35.5)	29 (45.3)	
Sex				
Male	171	128 (64.0)	43 (67.2)	0.895
Female	93	72 (36.0)	21 (32.8)	
Primary tumor location				
Colon	163	126 (63.0)	37 (57.8)	0.457
Rectum	101	74 (37.0)	27 (42.8)	
Primary tumor differentiation				
Well to moderate	206	155 (77.5)	51 (79.7)	0.713
Poor	58	45 (22.5)	13 (20.3)	
T stage^a^				
1–3	157	122 (65.2)	35 (66.5)	0.707
4	86	65 (34.8)	21 (33.5)	
N stage^b^				
0	103	78 (42.9)	25 (45.3)	0.814
1–2	135	104 (57.1)	31 (55.4)	
Timing of metastasis				
Synchronous	171	136 (68.0)	35 (54.7)	0.052
Metachronous	93	64 (32.0)	29 (45.3)	
Number of metastatic tumors				
1	140	102 (51.0)	38 (59.4)	0.501
2–3	99	78 (39.0)	21 (32.8)	
4–5	25	20 (10.0)	5 (7.8)	
Metastases diameter (cm)^c^				
≤ 3	173	134 (68.0)	39 (61.9)	0.371
> 3	87	63 (32.0)	24 (38.1)	
Preoperative CEA (ng/mL)^d^				
≤ 50	200	156 (81.7)	44 (73.3)	0.161
> 50	51	35 (18.3)	16 (26.7)	
Preoperative CA19-9 (U/mL)^a^				
≤ 35	166	128 (68.8)	38 (66.7)	0.760
> 35	77	58 (31.2)	19 (33.3)	
Preoperative chemotherapy				
Yes	122	98 (49.0)	24 (37.5)	0.108
No	142	102 (51.0)	40 (62.5)	
MSKCC-CRS^e^				
0–2	162	127 (73.4)	35 (67.3)	0.390
3–5	63	46 (26.6)	17 (32.7)	

*ACT* adjuvant chemotherapy, *CEA* carcinoembryonic antigen, *CA19-9* carbohydrate antigen 19-9, *MSKCC-CRS* Memorial Sloan-Kettering Cancer Center clinical risk score

^a^Data were available for 243 patients

^b^Data were available for 238 patients

^c^Data were available for 260 patients

^d^Data were available for 251 patients

^e^Data were available for 225 patients

### Relationships of ACT with clinicopathologic characteristics

The ACT and non-ACT groups did not significantly differ in clinicopathologic characteristics (Table 1), or in receiving NAC, or radiological tumor response (Table 2).

**Table 2:** Clinicopathologic characteristics of patients stratified by both neoadjuvant and adjuvant chemotherapy

Parameters	With preoperative chemotherapy (n = 122)		*P* value	Without preoperative chemotherapy (n = 142)		*P* value
	With ACT (n, %)	Without ACT (n, %)		With ACT (n, %)	Without ACT (n, %)	
Number of patients	98	24		102	40	
Age, years						
≤ 60	69 (70.4)	16 (66.7)	0.721	60 (58.8)	19 (47.5)	0.222
> 60	29 (29.6)	8 (33.3)		42 (41.2)	21 (52.5)	
Sex						
Male	65 (66.3)	17 (70.8)	0.673	63 (61.8)	26 (65.0)	0.720
Female	33 (33.7)	7 (29.2)		39 (38.2)	14 (35.0)	
Primary tumor location						
Colon	55 (56.1)	15 (62.5)	0.571	71 (69.6)	22 (55.0)	0.100
Rectum	43 (43.9)	9 (37.5)		31 (30.4)	18 (45.0)	
Primary tumor differentiation						
Well to moderate	72 (73.5)	20 (83.3)	0.315	83 (81.4)	31 (77.5)	0.602
Poor	26 (26.5)	4 (16.7)		19 (18.6)	9 (22.5)	
T stage^a^						
1–3	50 (53.2)	12 (54.5)	0.909	72 (77.4)	23 (67.6)	0.261
4	44 (46.8)	10 (45.5)		21 (22.6)	11 (32.4)	
N stage^b^						
0	41 (45.6)	12 (54.5)	0.449	37 (40.2)	13 (38.2)	0.840
1–2	49 (54.4)	10 (45.5)		55 (59.8)	21 (61.8)	
Timing of metastasis						
Synchronous	74 (75.5)	15 (62.5)	0.198	62 (60.8)	20 (50.0)	0.242
Metachronous	24 (24.5)	9 (37.5)		40 (39.2)	20 (50.0)	
Number of metastatic tumors						
1	29 (29.6)	9 (37.5)	0.453	73 (71.6)	29 (72.5)	0.912
2–5	69 (70.4)	15 (62.5)		29 (28.4)	11 (27.5)	
Metastases diameter (cm)^c^						
≤ 3	59 (62.1)	14 (58.3)	0.735	75 (73.5)	25 (64.1)	0.270
> 3	36 (37.9)	10 (41.7)		27 (26.5)	14 (35.9)	
Preoperative CEA (ng/mL)^d^						
≤ 50	78 (82.1)	17 (81.0)	0.901	78 (81.3)	27 (69.2)	0.128
> 50	17 (17.9)	4 (19.0)		18 (18.8)	12 (30.8)	
Preoperative CA19-9 (U/mL)^e^						
≤ 35	70 (76.9)	15 (75.0)	1.000	58 (61.1)	23 (62.2)	0.906
> 35	21 (23.1)	5 (25.0)		37 (38.9)	14 (37.8)	
MSKCC-CRS^f^						
0–2	54 (63.5)	9 (47.4)	0.193	73 (83.0)	26 (78.8)	0.597
3–5	31 (36.5)	10 (52.6)		15 (17.0)	7 (21.2)	
Preoperative chemotherapy regimen						
FOLFOX + XELOX	61 (62.2)	18 (75.0)	0.503			
FOLFIRI	31 (31.6)	5 (20.8)				
Capecitabine	6 (6.1)	1 (4.2)				
Radiological response to preoperative chemotherapy^g^						
PR	57 (58.8)	13 (54.2)	0.683			
SD	30 (30.9)	7 (29.2)				
PD	10 (10.3)	4 (16.7)				

*ACT* adjuvant chemotherapy, *CEA* carcinoembryonic antigen, *CA19-9* carbohydrate antigen 19-9, *MSKCC-CRS* Memorial Sloan-Kettering Cancer Center clinical risk score, *PD* progressive disease, *PR* partial response, *SD* stable disease

^a^Data were available for 243 patients

^b^Data were available for 238 patients

^c^Data were available for 260 patients

^d^Data were available for 251 patients

^e^Data were available for 243 patients

^f^Data were available for 225 patients

^g^Data were available for 121 patients

### Effect of ACT on survival outcomes

After their primary liver resections, all patients were followed up for a median of 35.5 months (range 2.0–126.0 months). Median follow-up time did not significantly differ between the ACT group (36.2 months; range 2.0–126.0 months) and the non-ACT group (30.5 months; range 2.0–117.0 months; *P* = 0.315; Table 3). Overall, 157 (59.5%) patients experienced tumor recurrence, and 104 (39.4%) patients died of tumor progression. The ACT and non-ACT groups did not significantly differ in postoperative recurrence (60.5% vs. 56.3%, *P* = 0.547), early recurrence (24.8% vs. 27.8%, *P* = 0.718), or the most common recurrence pattern—intrahepatic metastasis (51.0% vs. 60.9%, *P* = 0.389).

**Table 3:** Postoperative recurrence in patients with colorectal oligometastases to liver after curative liver resection, with or without adjuvant chemotherapy

Parameters	With ACT (n, %)	Without ACT (n, %)	*P* value
Postoperative recurrence (n = 264)			
Yes	121 (60.5)	36 (56.2)	0.547
No	79 (39.5)	28 (43.8)	
Recurrence period (n = 157)			
Early recurrence	30 (24.8)	10 (27.8)	0.718
Latter recurrence	91 (75.2)	26 (72.2)	
Recurrence pattern (n = 127)^a^			
Intrahepatic metastases	53 (51.0)	14 (60.9)	0.389
Extrahepatic metastases	51 (49.0)	9 (39.1)	

*ACT* adjuvant chemotherapy

^a^Data of recurrence pattern were unavailable for 30 patients

**Fig. 1:**
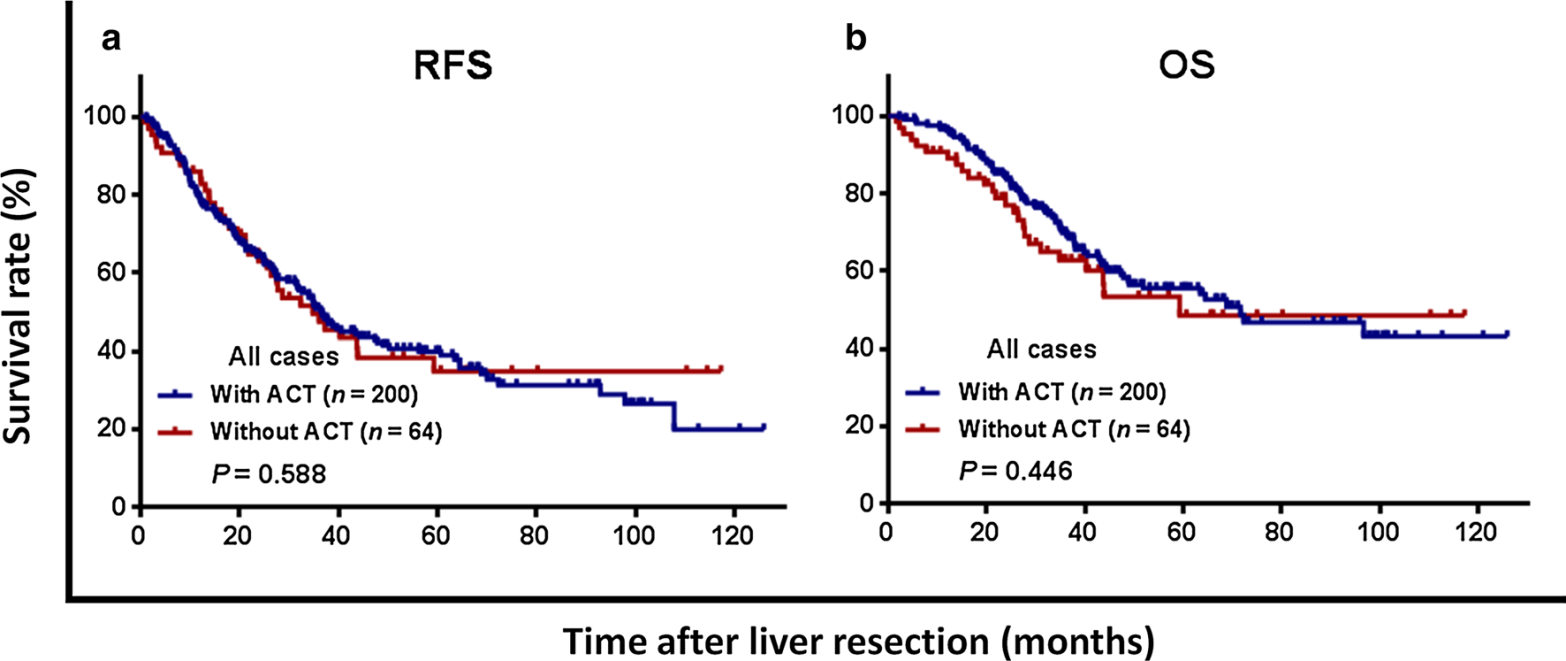
Kaplan–Meier survival curves comparing 3-year (**a**) recurrence-free survival (RFS) and (**b**) overall survival (OS) rates, based on administration of adjuvant chemotherapy (ACT) in patients who develop colorectal oligometastases to liver after curative liver resection

**Fig. 2:**
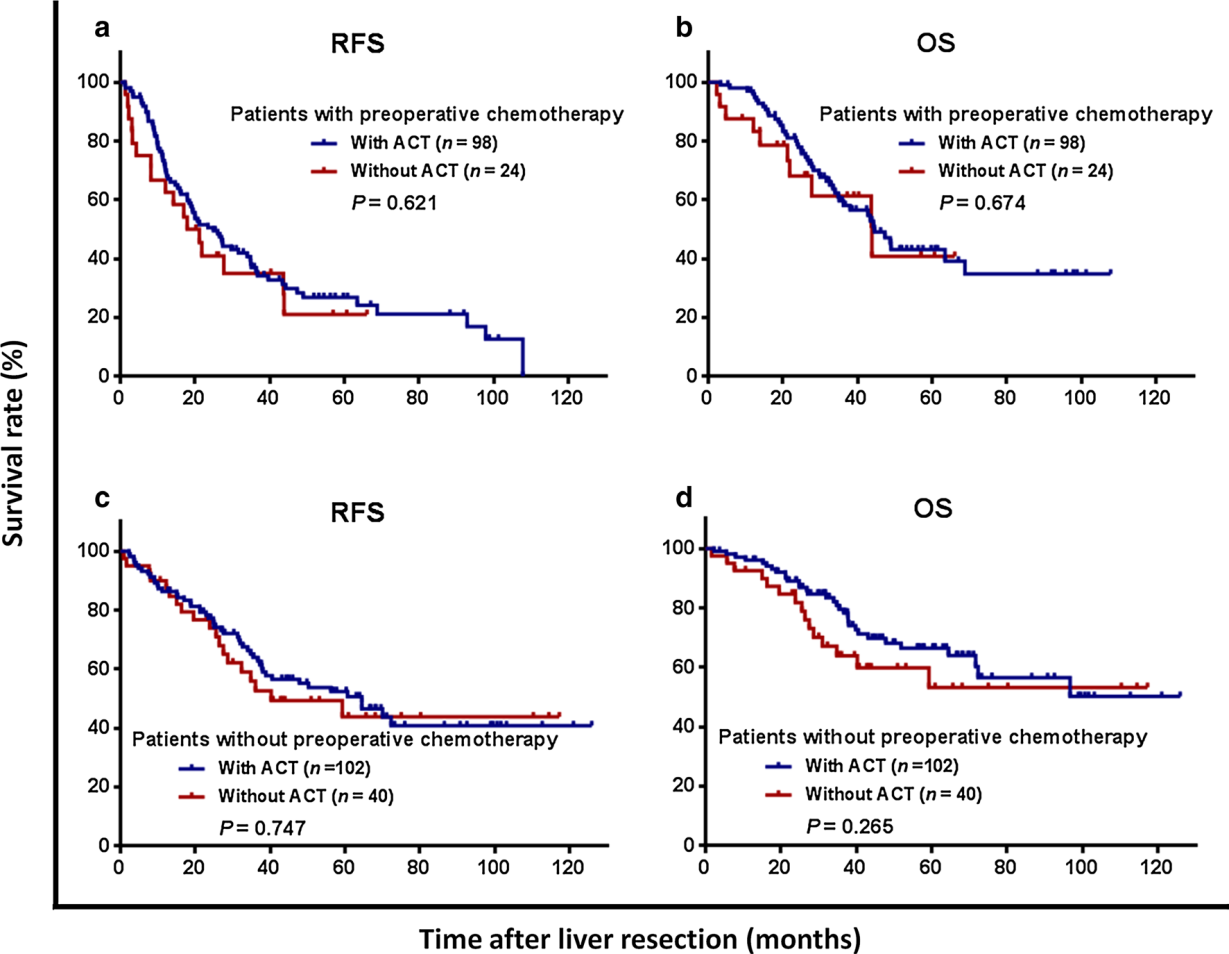
Kaplan–Meier survival curves of patients with or without neoadjuvant chemotherapy (NAC) stratified by the administration of adjuvant chemotherapy (ACT). **a** Recurrence-free survival (RFS) in the NAC group; **b** overall survival (OS) in the NAC group; **c** RFS in the non-NAC group; and **d** OS in the non-NAC group

Three-year RFS was 43.0%, and OS was 68.1%, for the entire cohort, and did not significantly differ between the ACT and non-ACT groups (RFS: 42.1% vs. 45.7%, *P *= 0.588, Fig. 1a; OS: 69.7% vs. 62.7%, *P *= 0.446, Fig. 1b). Among patients who received NAC, 3-year RFS and OS rates were not significantly different between the ACT and non-ACT groups (RFS: 27.5% vs. 35.1%, *P *= 0.621, Fig. 2a; OS: 59.6% vs. 61.2%, *P *= 0.674, Fig. 2b). Likewise, 3-year RFS and OS rates were also comparable between the ACT and non-ACT groups in the absence of NAC (RFS: 56.1% vs. 52.0%, *P *= 0.747, Fig. 2c; OS: 79.5% vs. 63.8%, *P *= 0.265, Fig. 2d).

**Fig. 3:**
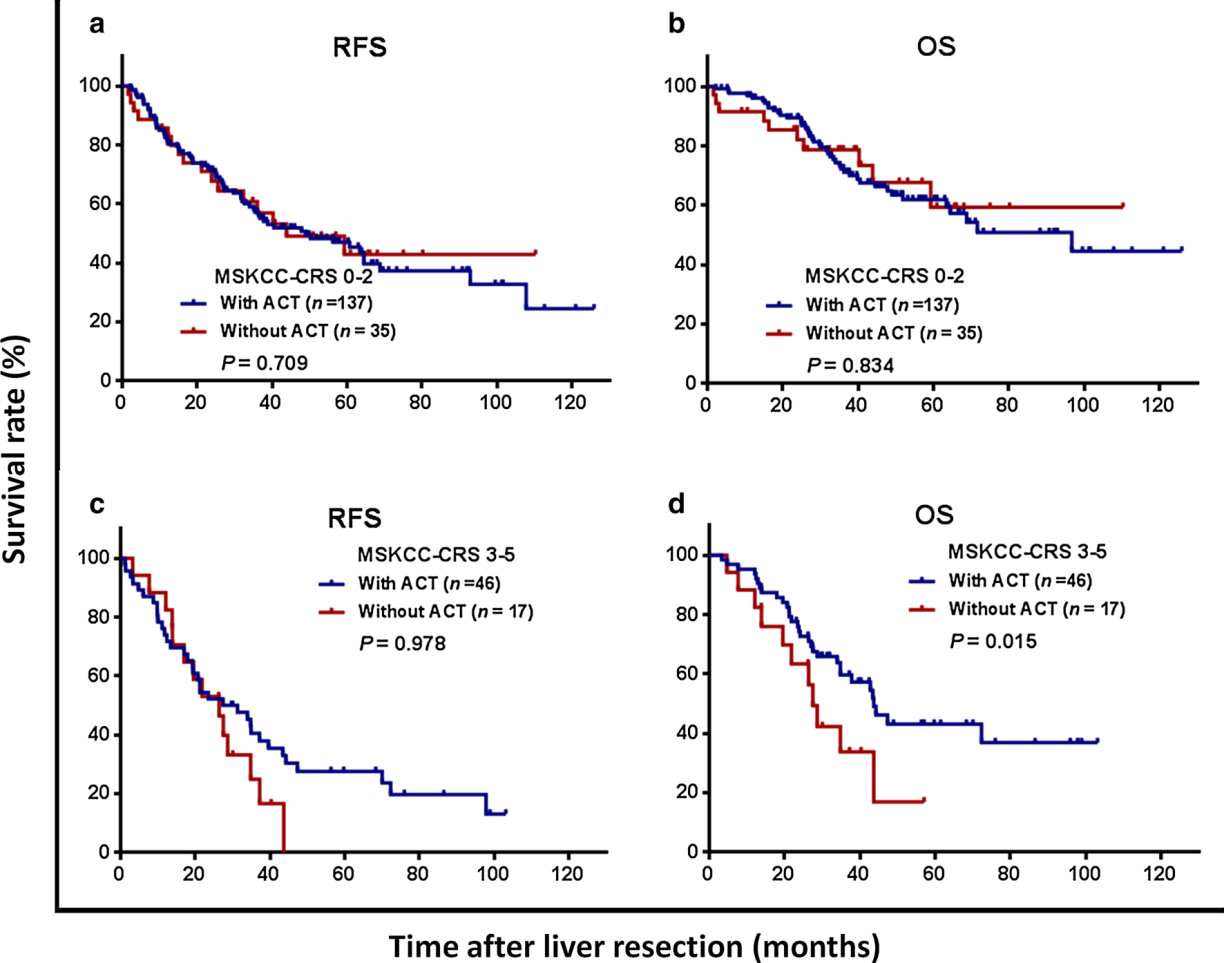
Kaplan–Meier survival curves of patients with lower risk (Memorial Sloan-Kettering Cancer Center clinical risk score [MSKCC-CRS] of 0–2) or higher risk (MSKCC-CRS 3–5) for chemotherapy, stratified by the administration of adjuvant chemotherapy (ACT). **a** Recurrence-free survival (RFS) in the lower-risk group; **b** overall survival (OS) in the lower-risk group 2; **c** RFS in the higher risk group; and **d** OS in the higher risk group

The patients were further stratified as high risk for recurrence (MSKCC-CRS 0–2) or low risk (MSKCC-CRS 3–5). Among the low-risk patients, 3-year DFS and OS rates were comparable between the ACT and non-ACT groups (RFS: 50.5% vs. 55.8%, *P* = 0.709, Fig. 3a; OS: 72.2% vs. 78.6% *P* = 0.834, Fig. 3b). Among the high-risk patients, although no significant difference was found in 3-year DFS rates (25.4% vs. 21.2%, *P* = 0.978, Fig. 3c), the 3-year OS rate was significantly higher in the ACT group than in the non-ACT group (68.2% vs. 33.8%, *P* = 0.015, Fig. 3d).

Among high-risk patients, univariate analysis associated ACT with a higher 3-year OS rate (HR: 0.402; 95% CI 0.188–0.858; *P *= 0.018; Table 4); and multivariate analysis showed ACT (HR: 0.350; 95% CI 0.161–0.759; *P *= 0.008) and T stage (HR: 2.247; 95% CI 1.093–4.622; *P *= 0.028) to be independent predictors of higher 3-year OS rates.

**Table 4:** Univariate and multivariate analyses of prognostic factors for overall survival in patients with MSKCC-CRS 3–5

Variable	Univariate		Multivariate	
	HR (95% CI)	*P* value	HR (95% CI)	*P* value
Age (≤ 60 years vs. > 60 years)	0.945 (0.457–1.955)	0.880		
Sex (male vs. female)	1.014 (0.492–2.090)	0.970		
Primary tumor location (rectum vs. colon)	1.562 (0.754–3.236)	0.230		
Primary tumor differentiation (poor vs. well to moderate)	1.601 (0.732–3.502)	0.160		
T stage (4 vs. 1–3)	1.957 (0.964–3.973)	0.063	2.247 (1.093–4.622)	0.028
N stage (positive vs. negative)	1.139 (0.345–3.760)	0.831		
Timing of metastasis (synchronous vs. metachronous)	1.190 (0.456–3.106)	0.722		
Number of metastatic tumors (> 1 vs. 1)	0.930 (0.279–3.095)	0.906		
Metastases diameter( > 3 cm vs. ≤ 3 cm)	1.625 (0.795–3.321)	0.183		
Preoperative CEA (> 50 ng/mL vs. ≤ 50 ng/mL)	0.727 (0.334–1.585)	0.423		
Preoperative CA19-9 (> 35 U/mL vs. ≤ 35 U/mL)	0.972 (0.444–2.129)	0.943		
Preoperative chemotherapy (yes vs. no)	1.470 (0.687–3.148)	0.321		
ACT (yes vs. no)	0.402 (0.188–0.858)	0.018	0.350 (0.161–0.759)	0.008

*ACT* adjuvant chemotherapy, *CA19-9* carbohydrate antigen 19-9, *CEA* carcinoembryonic antigen, *CI* confidence interval, *HR* hazard ratio, *MSKCC-CRS* Memorial Sloan-Kettering Cancer Center clinical risk score

## Discussion

The efficacy of ACT in prolonging survival of patients after curative resection of CRC liver metastases remains unknown, especially in patients with CLO, who could potentially achieve longer survival after curative treatment. As evidence of whether ACT after curative liver resection is worthwhile is lacking, we investigated the role of ACT in patients with CLO after curative liver resection. Although we saw no significant benefit from ACT on RFS and OS (irrespective of NAC), it notably improved OS in high-risk patient.

Based on the beneficial effects of ACT on patients with resected stage III colon cancer [19, 20], several studies have assessed its efficacy in eliminating micrometastatic disease in patients with CRC liver metastases after liver resection. The EORTC trial 40983 first showed that perioperative chemotherapy with FOLFOX4 (folinic acid, 5-FU, and oxaliplatin) improved 3-year progression-free survival (PFS) in patients with initially resectable CRC liver metastases who underwent liver resection, compared with surgery alone (HR: 0.73, 95% CI 0.55–0.97, *P *= 0.025) [21]. After a median follow-up of 8.5 years in EORTC trial 40983, the 5-year OS rate was slightly higher in the perioperative chemotherapy group than in the surgery-alone group, but not significantly so (5-year OS rate: 57.3% vs. 54.4%, *P *= 0.350) [7]. However, the potential benefits of NAC and ACT could not be discerned in that setting. Although an analysis of pooled data from the EORTC 40923 trial and FFCD trial 9002 showed potential improvement in the 5-year RFS (36.7% vs. 27.7%, *P* = 0.058) and OS (52.8% vs. 39.6%, *P *= 0.095) in response to ACT with a 5-FU bolus-based regimen in patients after complete resection of CRC liver metastases, the differences between the ACT group and surgery-alone group were non-significant [11]. In the current study, differences between the ACT and non-ACT groups in 3-year RFS (42.1% vs. 45.7%, *P *= 0.588) and OS (69.7% vs. 62.7%, *P *= 0.446) among patients with CLO were smaller than those in the pooled analysis. In addition, ACT did not significantly decrease the rate of postoperative recurrence or affect the recurrence pattern. The selected group of patients in our study experienced favorable survival outcomes after liver resection irrespective of ACT administration (3-year RFS: 43.0% and 3-year OS rates: 68.1%), which implies that ACT has no effect on long-term survival.

NAC has been shown to benefit some patients by allowing conversion to stable or resectable disease, which can translate into better long-term survival [22, 23]. Here, we explored the effect of ACT, with or without NAC, on patient survival. No significant differences were observed in the 3-year RFS and OS between the ACT and non-ACT groups, with or without NAC. Thus, ACT did not provide a survival benefit, irrespective of NAC. Contrary to our results, a recent study by Wang et al. [24] demonstrated the efficacy of ACT in patients with CRC liver metastases who received NAC and liver resection, and suggested that ACT was an effective postoperative management strategy. Notably, the discrepancy in the results between the present study and the study by Wang et al. may be attributable to patient selection and the type of liver resection. In our study, only 28.0% of patients were classified as high-risk group, based on a limited number of liver metastasis, while in the study by Wang et al. 43.3% of patients were identified as high risk. In addition, 23.9% of patients underwent R1 resection in the study by Wang et al. Therefore, these data preliminarily indicate that the benefit of ACT might mainly depend on patients’ risk factors, but not on their acceptance of NAC.

Subgroup analyses based on various risk factors showed that ACT may not be the best treatment strategy for all patients with CLO. The efficacy of ACT was mainly observed in high-risk patients; ACT failed to prolong survival of patients with low risk of recurrence, which was consistent with many previous studies. A study by Rahbari et al. [25] demonstrated that ACT markedly improved survival in high-risk patients with MSKCC-CRS > 2 (HR: 0.40; 95% CI 0.23–0.69, *P* = 0.001), but failed to provide any benefit to patients with a MSKCC-CRS ≤ 2 (HR: 0.90; 95% CI 0.57–1.43, *P* = 0.670). Likewise, ACT provided no benefit for 5-year DFS (55.7% vs. 62.7%, *P* = 0.93) or OS (81.1% vs. 71.7%, *P* = 0.460) in patients with low MSKCC-CRS in the study by Nakai et al. [26]. Interestingly, by examining baseline parameters that predicted beneficial effects of perioperative chemotherapy on PFS in the EORTC 40983 trial, Sorbye et al. [27] demonstrated that patients with higher CEA levels (> 5 ng/mL) had better 3-year PFS than did patients who were treated with surgery alone (35% vs. 20%, *P* = 0.002). Hirokawa et al. [28] also found that ACT increased OS and RFS in 110 patients with CRC liver metastases who underwent initial liver resections and had > 2 risk factors, including H2 classification, invasive tumors (pT4), and positive lymph nodes. Based on the overall results of the study, early engagement by a MDT was needed to carefully evaluate patients’ risk status before receiving ACT, to increase chances of cure [29]. Thus, ACT could be considered a standard treatment strategy after liver resection for high-risk patients, but not for low-risk patients.

The present study had some limitations. First, this retrospective study employed an uncontrolled methodology with a limited number of patients from a single institution. Therefore, the findings need to be validated in a prospective study with a larger sample size. Second, the various ACT regimens and durations might have exerted specific prognostic effects that were not analyzed in the current study [30, 31]. Third, the short follow-up periods were insufficient to evaluate 5-year survival outcomes, and may also have led to underestimation of the effect of ACT on long-term survival. Moreover, the effect of microsatellite instability, and mutations on such biomarkers as KRAS proto-oncogene, NRAS proto-oncogene, B-Raf proto-oncogene, and phosphatidylinositol-4,5-bisphosphate 3-kinase catalytic subunit alpha on the efficacy of ACT was not assessed in the present study. Future studies should examine these biomarkers. Despite these limitations, our study shows a basis for further clinical trials to evaluate the efficacy of ACT in patients with CLO after curative resection.

## Conclusion

ACT provides prognostic benefits in high-risk patients, but not low-risk patients, who develop CLO after undergoing curative liver resection. To optimize use of ACT, patients’ risk status should be determined while forming early management plans. Further prospective studies are warranted to validate our results.

## Abbreviations

5-FU: 5-fluorouracil
ACT: adjuvant chemotherapy
CA-19-9: carbohydrate antigen-19-9
CEA: carcinoembryonic antigen
CI: confidence interval
CLO: colorectal liver oligometastases
CRC: colorectal cancer
CT: computerized tomography
HR: hazard ratio
LV: leucovorin
MDT: multidisciplinary team
MRI: magnetic resonance imaging
MSKCC-CRS: Memorial Sloan-Kettering Cancer Center clinical risk scores
NAC: neoadjuvant chemotherapy
OS: overall survival
RFS: recurrence-free survival

## References

[1] O’Reilly DA, Poston GJ (2006). Colorectal liver metastases: current and future perspectives. Future Oncol..

[2] Van Cutsem E, Cervantes A, Adam R, Sobrero A, Van Krieken JH, Aderka D (2016). ESMO consensus guidelines for the management of patients with metastatic colorectal cancer. Ann Oncol.

[3] Dexiang Z, Li R, Ye W, Haifu W, Yunshi Z, Qinghai Y (2012). Outcome of patients with colorectal liver metastasis: analysis of 1613 consecutive cases. Ann Surg Oncol.

[4] Sadot E, Groot KB, Leal JN, Shia J, Gonen M, Allen PJ (2015). Resection margin and survival in 2368 patients undergoing hepatic resection for metastatic colorectal cancer: surgical technique or biologic surrogate?. Ann Surg.

[5] Chan KM, Wu TH, Cheng CH, Lee WC, Chiang JM, Chen JS (2014). Prognostic significance of the number of tumors and aggressive surgical approach in colorectal cancer hepatic metastasis. World J Surg Oncol..

[6] D’Angelica M, Kornprat P, Gonen M, DeMatteo RP, Fong Y, Blumgart LH (2011). Effect on outcome of recurrence patterns after hepatectomy for colorectal metastases. Ann Surg Oncol.

[7] Nordlinger B, Sorbye H, Glimelius B, Poston GJ, Schlag PM, Rougier P (2013). Perioperative FOLFOX4 chemotherapy and surgery versus surgery alone for resectable liver metastases from colorectal cancer (EORTC 40983): long-term results of a randomised, controlled, phase 3 trial. Lancet Oncol..

[8] Parks R, Gonen M, Kemeny N, Jarnagin W, D’Angelica M, DeMatteo R (2007). Adjuvant chemotherapy improves survival after resection of hepatic colorectal metastases: analysis of data from two continents. J Am Coll Surg.

[9] Kim HR, Min BS, Kim JS, Shin SJ, Ahn JB, Rho JK (2011). Efficacy of oxaliplatin-based chemotherapy in curatively resected colorectal cancer with liver metastasis. Oncology-Basel.

[10] Lu Z, Peng J, Wang Z, Pan Z, Yuan Y, Wan D (2016). High preoperative serum CA19-9 level is predictive of poor prognosis for patients with colorectal liver oligometastases undergoing hepatic resection. Med Oncol.

[11] Mitry E, Fields AL, Bleiberg H, Labianca R, Portier G, Tu D (2008). Adjuvant chemotherapy after potentially curative resection of metastases from colorectal cancer: a pooled analysis of two randomized trials. J Clin Oncol.

[12] Lin HH, Chang YY, Lin JK, Jiang JK, Lin CC, Lan YT (2014). The role of adjuvant chemotherapy in stage II colorectal cancer patients. Int J Colorectal Dis.

[13] Ooki A, Akagi K, Yatsuoka T, Asayama M, Hara H, Nishimura Y (2017). Lymph node ratio as a risk factor for recurrence after adjuvant chemotherapy in stage III colorectal cancer. J Gastrointest Surg..

[14] Fong Y, Fortner J, Sun RL, Brennan MF, Blumgart LH (1999). Clinical score for predicting recurrence after hepatic resection for metastatic colorectal cancer: analysis of 1001 consecutive cases. Ann Surg.

[15] Peng J, Li H, Ou Q, Lin J, Wu X, Lu Z (2017). Preoperative lymphocyte-to-monocyte ratio represents a superior predictor compared with neutrophil-to-lymphocyte and platelet-to-lymphocyte ratios for colorectal liver-only metastases survival. Onco Targets Ther..

[16] Eisenhauer EA, Therasse P, Bogaerts J, Schwartz LH, Sargent D, Ford R (2009). New response evaluation criteria in solid tumours: revised RECIST guideline (version 1.1). Eur J Cancer.

[17] Jung SW, Kim DS, Yu YD, Han JH, Suh SO (2016). Risk factors for cancer recurrence or death within 6 months after liver resection in patients with colorectal cancer liver metastasis. Ann Surg Treat Res..

[18] Malik HZ, Gomez D, Wong V, Al-Mukthar A, Toogood GJ, Lodge JP (2007). Predictors of early disease recurrence following hepatic resection for colorectal cancer metastasis. Eur J Surg Oncol.

[19] Haller DG, Tabernero J, Maroun J, de Braud F, Price T, Van Cutsem E (2011). Capecitabine plus oxaliplatin compared with fluorouracil and folinic acid as adjuvant therapy for stage III colon cancer. J Clin Oncol.

[20] Andre T, Boni C, Navarro M, Tabernero J, Hickish T, Topham C (2009). Improved overall survival with oxaliplatin, fluorouracil, and leucovorin as adjuvant treatment in stage II or III colon cancer in the MOSAIC trial. J Clin Oncol.

[21] Nordlinger B, Sorbye H, Glimelius B, Poston GJ, Schlag PM, Rougier P (2008). Perioperative chemotherapy with FOLFOX4 and surgery versus surgery alone for resectable liver metastases from colorectal cancer (EORTC Intergroup trial 40983): a randomised controlled trial. Lancet.

[22] Cremolini C, Loupakis F, Antoniotti C, Lonardi S, Masi G, Salvatore L (2015). Early tumor shrinkage and depth of response predict long-term outcome in metastatic colorectal cancer patients treated with first-line chemotherapy plus bevacizumab: results from phase III TRIBE trial by the Gruppo Oncologico del Nord Ovest. Ann Oncol.

[23] Folprecht G, Gruenberger T, Bechstein WO, Raab HR, Lordick F, Hartmann JT (2010). Tumour response and secondary resectability of colorectal liver metastases following neoadjuvant chemotherapy with cetuximab: the CELIM randomised phase 2 trial. Lancet Oncol..

[24] Wang Y, Wang ZQ, Wang FH, Yuan YF, Li BK, Ding PR (2017). The role of adjuvant chemotherapy for colorectal liver metastasectomy after pre-operative chemotherapy: is the treatment worthwhile?. J Cancer..

[25] Rahbari NN, Reissfelder C, Schulze-Bergkamen H, Jager D, Buchler MW, Weitz J (2014). Adjuvant therapy after resection of colorectal liver metastases: the predictive value of the MSKCC clinical risk score in the era of modern chemotherapy. BMC Cancer..

[26] Nakai T, Ishikawa H, Tokoro T, Okuno K (2015). The clinical risk score predicts the effectiveness of adjuvant chemotherapy for colorectal liver metastasis. World J Surg.

[27] Sorbye H, Mauer M, Gruenberger T, Glimelius B, Poston GJ, Schlag PM (2012). Predictive factors for the benefit of perioperative FOLFOX for resectable liver metastasis in colorectal cancer patients (EORTC Intergroup Trial 40983). Ann Surg.

[28] Hirokawa F, Hayashi M, Miyamoto Y, Asakuma M, Shimizu T, Komeda K (2014). Reconsideration of the indications for adjuvant chemotherapy for liver metastases from colorectal cancer after initial hepatectomy. Ann Surg Oncol.

[29] Weiser MR, Jarnagin WR, Saltz LB (2013). Colorectal cancer patients with oligometastatic liver disease: what is the optimal approach?. Oncology (Williston Park)..

[30] Schmoll HJ, Tabernero J, Maroun J, de Braud F, Price T, Van Cutsem E (2015). Capecitabine plus oxaliplatin compared with fluorouracil/folinic acid as adjuvant therapy for stage III colon cancer: final results of the NO16968 randomized controlled phase III trial. J Clin Oncol.

[31] Neugut AI, Matasar M, Wang X, McBride R, Jacobson JS, Tsai WY (2006). Duration of adjuvant chemotherapy for colon cancer and survival among the elderly. J Clin Oncol.

